# Meta-Analysis and Systematic Review of the Association between a Hypoactive *NCF1* Variant and Various Autoimmune Diseases

**DOI:** 10.3390/antiox11081589

**Published:** 2022-08-16

**Authors:** Liang Zhang, Jacqueline Wax, Renliang Huang, Frank Petersen, Xinhua Yu

**Affiliations:** 1 Priority Area Chronic Lung Diseases, Research Center Borstel, Member of the German Center for Lung Research (DZL), 23845 Borstel, Germany; 2 Hainan Women and Children’s Medical Center, Haikou 571100, China

**Keywords:** neutrophil cytosolic factor 1, reactive oxygen species, genetic association

## Abstract

Genetic association studies have discovered the *GTF2I-NCF1* intergenic region as a strong susceptibility locus for multiple autoimmune disorders, with the missense mutation *NCF1* rs201802880 as the causal polymorphism. In this work, we aimed to perform a comprehensive meta-analysis of the association of the *GTF2I-NCF1* locus with various autoimmune diseases and to provide a systemic review on potential mechanisms underlying the effect of the causal *NCF1* risk variants. The frequencies of the two most extensively investigated polymorphisms within the locus, *GTF2I* rs117026326 and *NCF1* rs201802880, vary remarkably across the world, with the highest frequencies in East Asian populations. Meta-analysis showed that the *GTF2I-NCF1* locus is significantly associated with primary Sjögren’s syndrome, systemic lupus erythematosus, systemic sclerosis, and neuromyelitis optica spectrum disorder. The causal *NCF1* rs201802880 polymorphism leads to an amino acid substitution of p.Arg90His in the p47phox subunit of the phagocyte NADPH oxidase. The autoimmune disease risk His90 variant results in a reduced ROS production in phagocytes. Clinical and experimental evidence shows that the hypoactive His90 variant might contribute to the development of autoimmune disorders via multiple mechanisms, including impairing the clearance of apoptotic cells, regulating the mitochondria ROS-associated formation of neutrophil extracellular traps, promoting the activation and differentiation of autoreactive T cells, and enhancing type I IFN responses. In conclusion, the identification of the association of *NCF1* with autoimmune disorders demonstrates that ROS is an essential regulator of immune tolerance and autoimmunity mediated disease manifestations.

## 1. Introduction

Genome-wide association studies (GWAS) have revolutionized the dissection of the genetic basis of autoimmune disorders. GWAS often lead to the discovery of a large number of susceptibility loci, each of which shows a mild contribution to the disease. For example, multiple GWAS in the last 15 years have uncovered more than 200 genetic loci that are independently associated with multiple sclerosis, while none of them show a odds ratio (OR) of more than 1.5 [1,2]. In 2013, a strong susceptibility loci within the *GTF2I*–*NCF1* intergenic region at 7q11.23 was identified for primary Sjögren’s syndrome (pSS), with the *GTF2I* rs117026326 as the most significant polymorphism (OR = 2.20, *p* = 1.31 × 10^−53^) [3]. Subsequently, this strong association has been confirmed [4] and extended to other autoimmune disorders, including systemic lupus erythematosus (SLE) [5], systemic sclerosis (SSc) [6], and neuromyelitis optica spectrum disorder (NMOSD) [7].

Since the *GTF2I* rs117026326 is an intronic SNP, efforts have been made to identify the causal variant of this novel susceptibility locus for multiple autoimmune diseases. In 2017, two studies reported that a missense mutation in neutrophil cytosolic factor 1 (*NCF1*) within the locus is associated with SLE, pSS and rheumatoid arthritis (RA) [8,9]. The *NCF1* rs201802880 G > A polymorphism that is associated disease susceptibility, age at diagnosis but not disease activity in SLE leads to a shift from Arg to His at position 90 which is a evolutionarily conserved residue in the p47phox subunit of the phagocyte NADPH oxidase complex [8,10]. The disease risk His90 variant reduces the production of reactive oxygen species (ROS) and increases the expression of type 1 interferon (IFN-I)-regulated genes [8,9], suggesting it is a putative causal variant of the *GTF2I*–*NCF1* intergenic susceptibility locus. This notion was verified by experimental evidence obtained from NCF1-His90 knock-in (KI) mice [11]. Compared to wild type (WT) littermate controls, NCF1-His90 KI mice show a reduced ROS production, elevated type IFN-I scores, splenomegaly, and increased germinal center B cells and plasma cells. Moreover, NCF1-His90 KI mice but not WT littermate controls develop autoantibodies and SLE-like kidney pathology after challenge with pristane [11]. In the current study, we performed a comprehensive meta-analysis to evaluate the association of the *GTF2I*–*NCF1* intergenic susceptibility locus with various autoimmune diseases. In addition, we aimed to provide an overview of potential mechanisms underlying the role of the causal NCF1-His90 variant in autoimmune conditions.

## 2. Methods

### 2.1. Identification of Eligible Studies

To obtain an overview of the association between the *GTF2I*–*NCF1* locus and autoimmune diseases, we carried out a comprehensive meta-analysis. A search of the Medline database (https://www.ncbi.nlm.nih.gov/pubmed (accessed on 30 March 2022)) was performed to identify eligible studies. First of all, the keyword ‘rs117026326’ and ‘(Arg90His) OR (rs201802880)’ were used for the search without any limitations. In a second step, the full text of all articles from step one were reviewed to identify eligible studies. The following studies were excluded: (a) review articles or comment, (b) non-genetic association studies, and (c) genetic studies for non-autoimmune diseases.

### 2.2. Data Extraction

Data extraction was conducted as described previously [12]. Briefly, the following information was collected from each study: first author’s name, year of publication, the population of origin, type of autoimmune disease, the number of cases and controls, and frequencies of the rs117026326 and rs201802880 allele in both cases and controls. For studies including several case–control populations, each case–control population was extracted separately. Two participants searched whole the articles that needed to be extracted independently, and the extracted data were checked by a third participant.

### 2.3. Data Evaluation and Statistical Analysis

Cochran’s Q-statistics was applied to evaluate the heterogeneity across studies. The random-effect model was used for meta-analysis when heterogeneity was indicated by a significant Q-statistic (*p* < 0.10), otherwise the common/fixed effect model was used. By comparing frequencies of alleles, the odds ratio (OR), 95% confidence intervals (CI), and *p* values were estimated for each individual case–control study. Meta-analysis was performed to calculate the pooled OR, 95% CI and *p* values. The presence of publication bias was evaluated by examining the asymmetry of the funnel plot using Egger’s regression test. All statistical analyses were performed using the R software (version 4.1.2) and Comprehensive Meta-Analysis computer program (Biosta, Englewood, NJ, USA).

## 3. Results

### 3.1. Frequency Distribution of the GTF2I and NCF1 Polymorphisms across Populations

Notably, although the *GTF2I-NCF1* locus at 7q11.23 is strongly associated with pSS in Chinese [3], such an association has not been observed in Caucasians [13], suggesting that susceptibility variants within the locus might be remarkably less prevalent in Caucasians than in Chinese. This notion is supported by the frequency distribution of the *GTF2I* rs117026326 and *NCF1* rs201802880 polymorphisms across populations.

**Figure 1:**
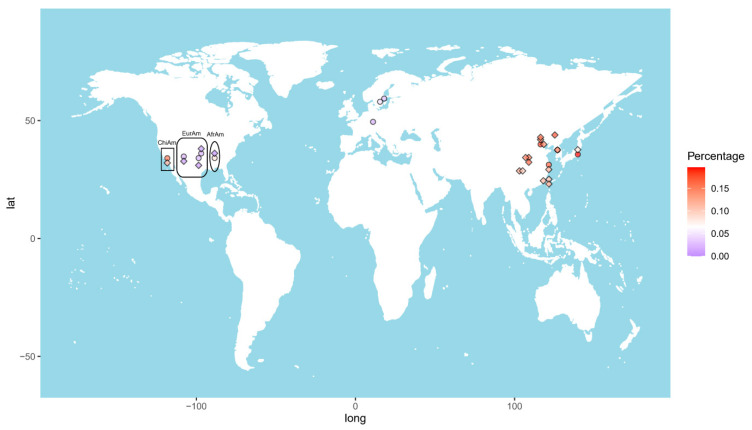
Heat map of the allele frequencies of the *GTF2I* rs117026326 (diamond) and the *NCF1* rs201802880 (circle) across the world. Each color dot represents frequency of the *GTF2I* rs117026326 or the *NCF1* rs201802880 in a single population. ChiAm: Chinese American; EurAm: European American; AfrAm: African American.

The allele frequency of the *GTF2I* rs117026326 polymorphism varies considerably across the world (Figure 1). The highest frequency (17.6%) was reported in Changchun, a city in Northeast China [7]. The frequency decreases toward the south and west China, showing 14.9% and 13.9% in Beijing [5,6], and 11.9% in Shanghai [9], 11.9% and 9.4% in Chengdu [14,15], 11.5% in Xiamen [4], and 10.4% in Taiwan [16]. This polymorphism is also prevalent in other Eastern Asia populations, with allele frequencies of 11.4% in Korean [9] and 8.0% in Japanese [17]. In line with this finding, the frequency in Chinese American in Los Angeles was reported to be 11.4% [9]. By contrast, those values in European American and African American are only 0.7% and 0.0%, respectively [9].

Due to highly homologous sequences among *NCF1* and its two pseudogenes, *NCF1B* and *NCF1C*, genotyping of the *NCF1* rs201802880 polymorphism is not possible with conventional methods [9]. To obtain the correct genotypes of the polymorphism, a NCF1-specific genomic fragment needs to be amplified and, subsequently, subjected to sequencing or TaqMan SNP Genotyping Assay [8,9,17]. The highest allele frequency of the *NCF1* rs201802880 polymorphism was reported in Japan (19.6%) [17], followed by Beijing, China (18.3%); Korea (18.1%); and Shanghai, China (16.7%). The frequency in Chinese American (15.6%) is considerably higher than in African American (8.2%) and European American (2.3%). As expected, the frequencies in Swedish (2.45%) and German (1.8%) populations are comparable to that in European American [8,18] (Figure 1).

Taken together, allele frequencies of *GTF2I* rs117026326 and *NCF1* rs201802880 polymorphisms are population dependent, with higher frequencies in East Asian populations than in others, which explains the strong association of the *GTF2I*-*NCF1* locus with autoimmune diseases in China, Korea, and Japan.

### 3.2. Meta-Analysis for the Association of the GTF2I-NCF1 Locus with Autoimmune Diseases

**Figure 2:**
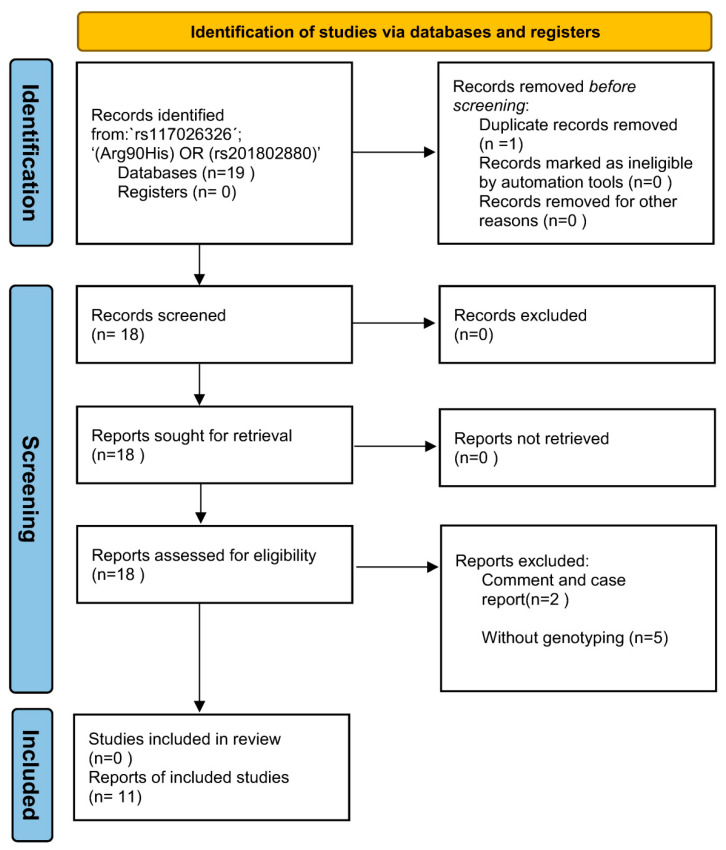
The PRISMA flow diagram of the meta-analysis.

**Figure 3:**
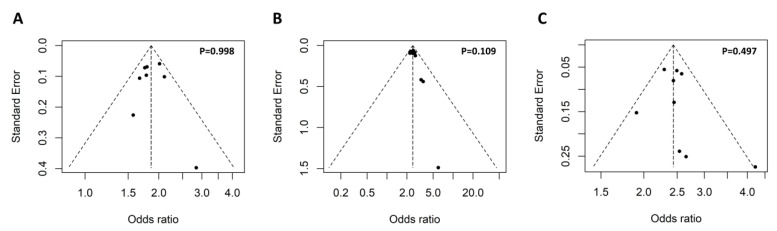
Symmetrical funnel plot used to assess publication bias. The funnel plot of the association of the *GTF2I* rs117026326 polymorphism with pSS (**A**) and SLE (**B**), as well as association of the *NCF1* rs201802880 polymorphism with SLE (**C**). Egger’s regression test was used to assess potential publication bias via funnel plot asymmetry, and a *p*-value less than 0.05 indicates presence publication bias.

The keyword ‘rs117026326´ and ‘(Arg90His) OR (rs201802880)’ were used for the search without any limitations, which identified 11 and 8 articles, respectively. Further review of the 19 articles led to the identification of 11 eligible studies for the meta-analysis (Figure 2). To access potential publication bias, the asymmetry of the funnel plot was examined. All *p* values of Egger’s regression test were >0.05, suggesting that there was no publication bias for the association of the two polymorphisms within the *GTF2I-NCF1* loci with pSS or SLE (Figure 3).

#### 3.2.1. Meta-Analysis for the Association of the GTF2I rs117026326 Polymorphism with Autoimmune Diseases

**Figure 4:**
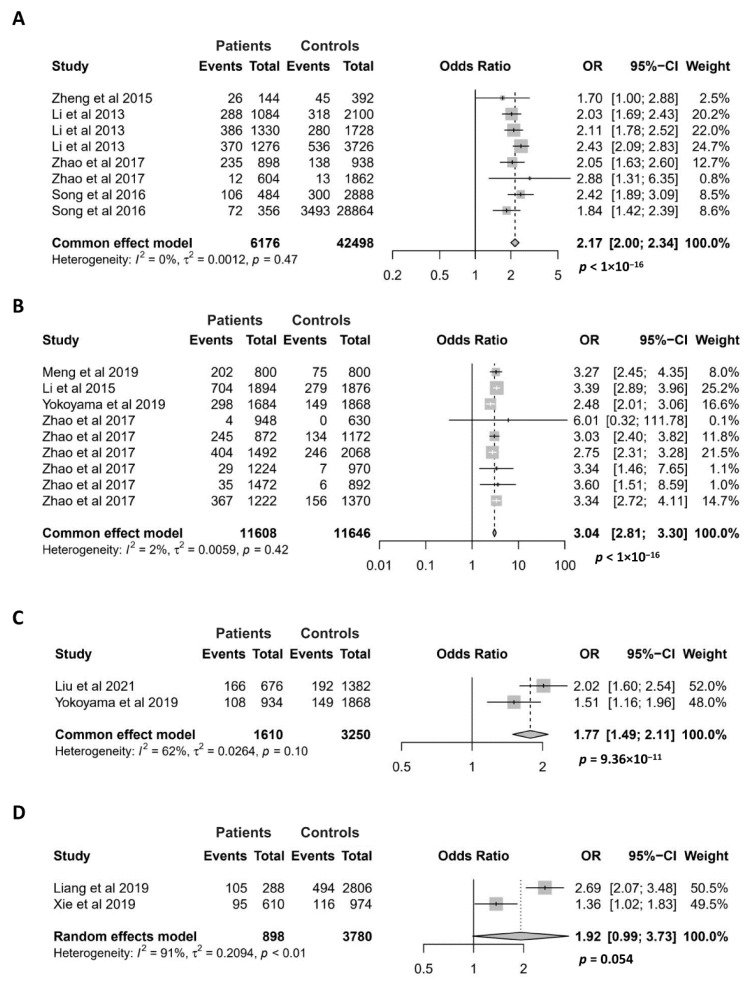
Association of the GTF2I rs117026326 polymorphism with pSS (**A**), SLE (**B**), SSc (**C**), and NMOSD (**D**). Data derived from the meta-analysis are presented as forest plot. Odds ratios (OR) and 95% confidence intervals (CI) of individual studies are represented by squares and horizontal lines, respectively. Size of the square represents the weight of the individual study in the meta-analysis. Diamonds represent the OR (center line of the respective diamond) and 95% CI (lateral tips of the respective diamond). The OR, 95% CI and *p* values for the meta-analysis are indicated by bold text. Information regarding individual case–control studies, including the first author name and year of publication, are indicated [3,4,5,6,7,9,14,15,16,17].

Since the *GTF2I* rs117026326 polymorphism exerts the strongest association with pSS in the discovery study of the susceptibility locus [3], this SNP has been extensively studied for its association with various autoimmune diseases, including pSS, SLE, SSc, NMOSD, multiple sclerosis (MS), and antineutrophil cytoplasmic antibody (ANCA)-associated vasculitides (AAV) [3,4,5,7,9,14,15,16,17]. The association of the *GTF2I* rs117026326 polymorphism with pSS, SLE, SSc and NMOSD has been investigated in at least two case–control studies, making them applicable for meta-analysis (Figure 4).

For the meta-analysis of the association between the *GTF2I* rs117026326 polymorphism and pSS, 8 case–control studies were recruited. All of them showed that the minor allele is associated with an increased risk of pSS [3,4,9,16]. As expected, our meta-analysis confirmed a highly significant association between the *GTF2I* rs117026326 polymorphism and pSS (OR = 2.17; 95% CI = 2.00–2.34, *p* < 1 × 10^−16^) (Figure 4A). The association between the GTF2I rs117026326 polymorphism and SLE has been investigated in 9 case–control studies, and 8 of them showed a significant association [5,9,14,17]. Our meta-analysis showed a strong association of the *GTF2I* rs117026326 polymorphism with SLE, where the minor allele conferred an approximate 3-fold risk for the disease (OR = 3.04; 95% CI = 2.81–3.30, *p* < 1 × 10^−16^) (Figure 4B). Meta-analysis also revealed a significant association between the *GTF2I* rs117026326 polymorphism and SSc with two case–control studies [6,16] (OR = 1.77; 95% CI = 1.49–2.11, *p* = 9.36 × 10^−11^) (Figure 4C). The association between the *GTF2I* rs117026326 polymorphism and NMOSD has been investigated in two case–control studies where both have shown a significant association [7,15]. The meta-analysis with the two studies revealed a marginally significant association of the *GTF2I* rs117026326 polymorphism with NMOSD (OR = 1.92; 95% CI = 0.99–3.73, *p* = 0.054) (Figure 4D).

Unlike the four autoimmune diseases mentioned above, MS and AAV are reported not to be associated with the *GTF2I* rs117026326 polymorphism [7,17]. The difference between two autoimmune demyelinating diseases of the central nervous system, MS and NMOSD, in their association with *GTF2I* is supported by our recent studies reporting that the *GTF2I* rs73366469, another Taq-SNP of the intergenic locus, is associated with NMOSD, but not with MS, myelin oligodendrocyte glycoprotein-associated disorders or anti-*N*-Methyl-D-Aspartate receptor encephalitis [19,20].

#### 3.2.2. Meta-Analysis for the Association of the *NCF1* rs201802880 Polymorphism with Autoimmune Diseases

**Figure 5:**
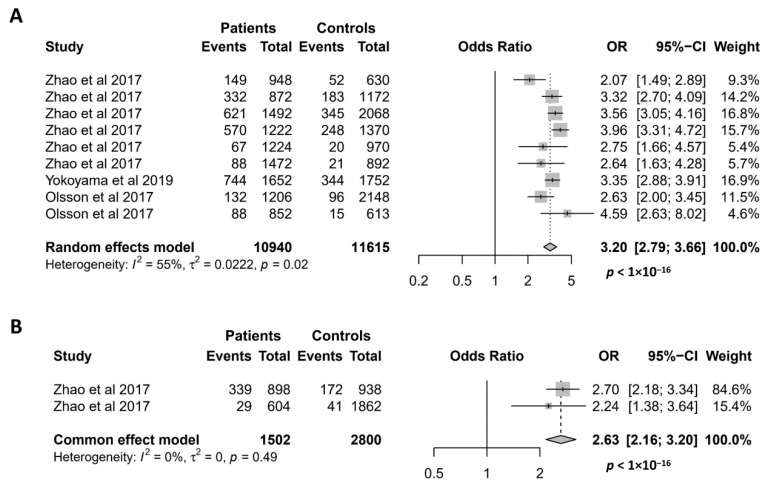
Association of the *NCF1* rs201802880 polymorphism with SLE (**A**) and pSS (**B**). Data derived from the meta-analysis are presented as forest plot. Odds ratios (OR) and 95% confidence intervals (CI) of individual studies are represented by squares and horizontal lines, respectively. Size of the square represents the weight of the individual study in the meta-analysis. Diamonds represent the OR (center line of the respective diamond) and 95% CI (lateral tips of the respective diamond). The OR, 95% CI and *p* values for the meta-analysis are indicated by bold text. Information regarding individual case–control studies, including the first author name and year of publication, are indicated [8,9,17].

Since it was discovered in 2017 [9], the missense mutation *NCF1* rs201802880 has been investigated for its association with 5 autoimmune conditions, namely SLE, pSS, RA, SSc, and AAV [8,9,17]. As shown in Figure 5A, the association of the *NCF1* rs201802880 polymorphism with SLE has been investigated in 9 case–control studies, and all of them reported that the minor allele is significantly associated with an increased risk of SLE [8,9,17]. Accordingly, our meta-analysis showed a strong association of the *NCF1* rs201802880 polymorphism with SLE, where the minor allele conferred a more than 3-fold risk of developing the disease (OR = 3.20; 95% CI = 2.79–3.66, *p* < 1 × 10^−16^). Meta-analysis of the association between the *NCF1* rs201802880 polymorphism and pSS was performed with two case–control studies [9]. The result showed a strong association of the mutant allele with an increased risk for pSS (OR = 2.63; 95% CI = 2.16–3.20, *p* < 1 × 10^−16^) (Figure 5B). Association of the *NCF1* rs201802880 polymorphism with other three autoimmune diseases was investigated in only one case–control study, where SSc and RA, but not AAV, are reported to be associated with the missense mutation [17].

## 4. Discussion

In the present study, we performed a meta-analysis demonstrating that the *GTF2I*-*NCF1* intergenic locus is associated with multiple autoimmune diseases, including pSS, SLE, SSc, and NMOSD. A major limitation of this study is the relative small number of studies retrieved for the analysis. Since the causal mutation within the *GTF2I-NCF1* locus was identified in 2017, its association with autoimmune diseases has not been extensively investigated. It is conceivable that some other autoimmune disorders are also associated with the susceptibility locus, and examination of the pattern of the association will help to understand the underlying mechanisms of the association.

Both clinical and experimental evidence have shown that the causal variation is the *NCF1* rs201802880 polymorphism which leads to the p.Arg90His substitution. *NCF1* encodes the p47phox which is one of five subunits of the NADPH oxidase 2 (NOX2), and the p.Arg90His residue is located in the phox domain which mediates binding of p47phox to the cellular membrane. It has been shown that the evolutionary conserved Arg90 residue is essentially involved in the binding to the cellular membrane and the NAPDH oxidase activity in response to various stimuli [21]. Compared to Arg90, the His90 variant reduces the ROS production and leads to an increased risk of developing multiple autoimmune diseases including SLE, pSS, SSc, and RA [8,9,22]. In accordance with these findings, *NCF1* variants that reduce NOX2-derived ROS production in rat and mice have also been shown to promote multiple experimental autoimmune diseases, such as pristane-induced arthritis, experimental autoimmune encephalomyelitis, and SLE-like disease [23,24,25]. The causal relationship between the human and rodent hypoactive *NCF1* mutations and various autoimmune disorders suggests an essential role of NOX2-derived ROS in the regulation of development of autoimmune disorders [26]. Clinical and experimental evidence have demonstrated that the NOX2-derived ROS might contribute to the pathogenesis of autoimmune diseases via multiple mechanisms.

### 4.1. Regulation of Apoptotic Cell Clearance

The phagocyte NOX2 complex is composed of two catalytic transmembrane subunits and three regulatory cytosolic subunits, including the p47phox encoded by *NCF1* [27]. ROS-reducing *NCF1* variants that lead to hypofunction of the NOX2 might impair the physiological function of phagocytes. Very recently, Geng and colleagues reported that bone marrow-derived macrophages from *NCF1* His90-KI mice result in an impaired efferocytosis [11]. Compared to WT controls, macrophages from the His90-KI mice show a decreased ROS production, reduced Hv1-dependent acidification, impaired maturation and proteolysis of phagosomes, which lead to a slower digestion of apoptotic cells [11]. This observation is in line with the finding that macrophages from mice carrying loss-of-function mutation in *gp91phox* encoding another NOX2 subunit show an impaired ability to clear apoptotic cells [28].

Under physiological condition, most tissue undergoes routine turnover of cells which mainly die via apoptosis, and the rapid removal of those apoptotic cells by phagocytes via efferocytosis is an essential step for the maintenance of tissue homeostasis. Defective clearance of apoptotic cells is associated with many pathological conditions, including autoimmune diseases, such as SLE [29]. For example, it has been shown that macrophages from patients with SLE are featured by a decreased phagocytic clearance of apoptotic cells [30,31]. Moreover, mice deficient in genes encoding molecules involved in apoptotic cell recognition and binding on the cell membrane of phagocytes develop spontaneously lupus-like disease [32,33,34]. Therefore, it is conceivable that the ROS-reducing *NCF1* variant causes a defective efferocytosis and thus impairs clearance of apoptotic cells, which further contributes to the development of SLE. This concept is supported by the observation that mice carrying hypoactive *NCF1* variant produce SLE-associated autoantibodies and develop chronic kidney inflammation spontaneously or after application of pristane [11,25]. In line with these findings, macrophages from SLE patients carrying the His90 variant show a decreased phagocytic ability compared to those from Arg90-carrying patients with SLE [11].

### 4.2. Regulation of Mitochondrial ROS-Associated NET Formation

As a crucial component in the first line of defense against micro-organisms, neutrophils are equipped with multiple anti-infective strategies, including phagocytosis, generation of ROS, proteases, and formation of neutrophil extracellular traps (NETs) [35]. Apart from their essential role in infection, neutrophils have been emerged as a player in the development of various autoimmune disorders [36].

In SLE, neutrophils from patients are characterized with multiple abnormalities in their functions, such as increased apoptosis, elevated release of NETs, aberrant NETosis, impaired phagocytosis, and enriched circulating low-density granulocytes [37,38,39,40], supporting a crucial role of these cells in the disease pathogenesis. Among the different neutrophil functions, the role of NET formation in the pathogenesis of SLE has been extensively investigated. As complex three-dimensional structures committed to trap circulating micropathogens, NETs are composed of chromosome DNA, histone, and cytoplasmic granule proteins, providing a potential resource of SLE autoantigens [41,42]. Despite extensive investigation, the role of NETs in SLE remains elucidative. On the one hand, increased NET release and impaired clearance of NET components have been observed in SLE [43,44] and immune complexes formed by autoantibodies and NET-derived antigen can stimulate the production of IFN-α [40] and activate autoreactive B cells [45], favoring disease-promoting role of NETs in SLE. On the other hand, studies using murine models of SLE have shown that NET deficiency can promote [46], inhibit [47] or have no impact [48] on lupus-like disease, suggesting that NETs are not an indispensible component but may contribute to the development of SLE depending on the respective pathological condition.

Neutrophils from patients with CGD are unable to produce NETs [49], highlighting an essential role of NOX2 activity in NET formation. Recently, Linge et al. reported that human neutrophils with the hypoactive *NCF1* His90 variant show a decreased NET formation in response to PMA stimulation, compared to Arg90 neutrophils [10]. Moreover, an increased dependence on mtROS in PMA-mediated NET formation has been observed in His90 neutrophils [10]. Apart from ROS produced via NOX2, neutrophil activation also leads to the generation of mitochondria-derived ROS (mtROS) [50]. When NOX2-derived ROS are impaired, mtROS are likely involved in the formation of NETs [50]. By contrast to the anti-inflammatory role of the NOX2-derived ROS, mtROS are potentially inflammatory because it causes oxidation of mitochondrial DNA [51,52,53], implicating that imbalance in the usage of NOX2-derived ROS and mtROS might affect autoimmune inflammatory processes. Therefore, it is likely that the involvement of mtROS in the formation of NETs in His90 neutrophils makes the NETs pathogenic. This concept is partially supported by evidence that mtROS-associated NETs containing mtDNA aggravates lupus-like disease in mice [51]. However, it needs to be further validated in future studies.

### 4.3. Regulation of T Cell Responses

Dysregulated autoreactive T cell responses have been observed in mice carrying His90 allele or other ROS-reducing variants [11,23,54], suggesting a role of autoimmune disease risk *NCF1* variants in the regulation of T cell homeostasis. Although T cells do not exert oxidative burst, multiple evidence show that *NCF1* mutation are able to regulate T cell homeostasis via antigen presenting cells (APCs).

As compared with WT mice, His90 KI mice are featured by increased ratios of splenic follicular T helper 2 (Tfh2) to either T follicular regulatory (Tfr) or Tfh1 cells [11], suggesting a role of the hypoactive His90 variant and reduced ROS in the regulation of differentiation of follicular T helpers. In line with this experimental observation, SLE patients carrying His90 variant have increased frequencies of circulating Tfh and Tfh2 cells but decreased frequencies of Tfh1 and Tfr cells [11]. Expanded Tfh2 cell populations are likely caused by the interaction between T cells and APCs with defective efferocytosis. Mechanistically, the His90 variant causes defective efferocytosis and persistence of apoptotic cell proteins within phagosomes of APCs, which leads to the enhanced presentation of an apoptotic cell-associated antigen with increased CD40 expression and consequent expansion of Tfh and Tfh2 cells [11,55].

Hypoactive *NCF1* variants also promote the expansion of autoreactive effector T helpers. In 2017, Sarelia and colleagues reported that KI mice carrying a ROS-reducing *NCF1* variant originated from rat, develop severe collagen induced arthritis (CIA) while mice expressing functional *NCF1* develop only mild symptoms [54], demonstrating a protective role of ROS in the development of autoimmune arthritis. Notably, the hypoactive *NCF1* variant promotes the expansion of immunodominant collagen II-specific Th17 cells [54] which are a major contributor to the pathogenesis of CIA [56]. The effect of promoting the expansion of collagen II-specific CD4 effector T cells has also been reported in mice expressing another hypoactive *NCF1* variant [23]. A possible mechanism underlying the role of reduced ROS in regulation of CD4 effector T cells has been proposed by Gelderman and colleagues [57,58]. According to their hypothesis, APCs with lower capacity to produce ROS are associated with an increased number of reduced thiol groups (-SH) on membrane surfaces of T cells, which lowers the threshold for T cell reactivity and enhances proliferative responses [57,58].

In addition to the regulation of T helper cells, NOX2-derieved ROS is also involved in the induction of regulatory T cells (Tregs). Compared to controls with functional *NCF1*, animals carrying hypoactive *NCF1* variant show similar levels of naïve Tregs but a decreased levels of induced Tregs, suggesting that NOX2-derived ROS is require for the induction of Tregs [59]. This notion is supported by in vitro evidence that macrophages induce CD4^+^CD25^+^FoxP3^+^ Tregs in a ROS-dependent manner [59]. Taken together, the hypoactive *NCF1* variant might contribute to T cell autoimmunity by promoting autoreactive T helpers and inhibiting the induction of Tregs.

### 4.4. Regulation of Type 1 IFN Signaling

In 2014, Kelkka and colleagues reported that both chronic granulomatous disease (CGD) patients and mice lacking functional NOX2 complex exhibit a prominent type I interferon (IFN) response signature and elevated levels of autoantibodies [25], suggesting for the first time that type I IFN signaling as a potential mediator connecting ROS deficiency to autoimmunity. In line with this finding, the hypoactive *NCF1* His90 variant is associated with an increased expression of type 1 interferon-regulated genes in patients with autoimmune diseases, such as RA and SLE [8,10]. Furthermore, *NCF1* His90 KI mice show an elevated type I IFN response and SLE-associated autoantibodies compared to WT littermate controls [11]. Therefore, the prominent type I IFN response might be a potential contributor to the His90 variant-caused autoimmune conditions. The type I IFN response is committed to combat viral infection, and it can be induced by both environmental and endogenous factors [60,61]. By investigating germ-free *NCF1* mutated mice, Kelkka and colleagues showed that the upregulated type I IFN response associated with reduced ROS is of endogenous origin [25].

There is an increasing body of evidences that type I IFN response is associated with autoimmune conditions, especially autoimmune rheumatoid diseases, such as SLE, pSS, and RA [62,63]. Elevated levels of circulating IFN-α have been observed in patients with SLE and they are associated with disease activity and clinical manifestations [64]. In murine models of SLE, increased type I IFN response has been observed [65], and application of IFN-α increases immune complex deposition in the kidneys and consequently exacerbates the disease [66,67]. In addition to in SLE, unregulated expression of type I IFN and IFN-stimulated genes has been observed in pSS [68,69]. In addition, elevated IFN signature is regarded as a biomarker for disease activity and response to therapy in RA [70,71]. Type I IFNs are capable to promote multiple immunological processes, including maturation of myeloid DCs (mDCs), CD4 T helper cell differentiation, B cell activation, plasma cell differentiation, antibody production, and Ig class switching [72,73,74]. Specifically with regard to defective NCF1-caused type I IFN responses, it has been shown that hypoactive *NCF1* variant leads to elevated type I IFN responses and the expansion of germinal center (GC) B cells in mice [11]. Given that type I IFN is capable to induce B cells to express CD38 [75] that prevents apoptosis of GC B cells [76], and that B cells from CDG patients expressed higher levels of CD38 [25], it is conceivable that type I IFN response contributes to the NCF1-associated autoimmunity via inhibiting apoptosis of autoreactive GC B cells.

## 5. Conclusions

The meta-analysis in this study demonstrates that the *GTFI-NCF1* susceptibility locus is associated with multiple autoimmune diseases. The causal *NCF1* His90 variant leads to an impaired NOX2 activity and confers a risk for various autoimmune conditions. Experimental and clinical evidence suggests that the hypoactive *NCF1* His90 variant might contribute to autoimmune conditions via multiple pathways, including impairing apoptotic cell clearance, regulating the formation of N.

ETs, promoting the differentiation of autoreactive T cells, and increasing type I IFN responses. In addition, since NOX2-derived ROS play an important role in autophagy and aberrations of autophagy has been observed in autoimmune diseases, such as SLE [77,78], a potential mechanism underlying hypoactive *NCF1* variant-caused autoimmune conditions might be regulating the homeostasis of autophagy. Of note, the *NCF1* His90 variant might contribute to different autoimmune diseases via different mechanisms, e.g., impairment of apoptotic cell clearance likely contributes to SLE. Since NOX2 is expressed in all phagocytes, the hypoactive His90 variant might contribute to autoimmune diseases via other mechanisms which need to be explored in future studies. In addition, given that susceptibility polymorphisms may impact the treatment of disease [79], it is interesting to explore the association of *NCF1* His90 variant with response to treatment in autoimmune diseases. In conclusion, the identification of the autoimmune disease risk *NCF1* His90 variant demonstrates an essential role of NOX2-derived ROS in the development of autoimmune diseases.

## Data Availability

Not applicable.
